# Burden of diet-related chronic diseases in Chinese and Japanese adults attributable to dietary risk factors from 1990 to 2021: a systematic analysis of the Global Burden of Disease Study 2021

**DOI:** 10.3389/fnut.2024.1472451

**Published:** 2025-01-24

**Authors:** Minyan Wang, Huan Ma, Chu Qin, Oscar Onayi Mandizadza, Haojie Ni, Yun Shi, Conghua Ji

**Affiliations:** School of Public Health, Zhejiang Chinese Medical University, Hangzhou, Zhejiang, China

**Keywords:** chronic diseases, neoplasms, cardiovascular disease, diabetes, dietary risk factors, disease burden

## Abstract

**Background:**

Chronic diseases are a major cause of death, contributing significantly to the global disease burden. The growing aging population and chronic disease burden in China and Japan have a substantial impact on health outcomes. Dietary factors, as key modifiable elements, are particularly important. Therefore, we aimed to analyze and compare the impact of dietary factors on the burden of chronic diseases in China and Japan and to develop measures to reduce this burden.

**Methods:**

According to the WHO classification of chronic diseases, we selected cardiovascular diseases, neoplasms, and diabetes for analysis. We collected relevant data from the GBD database, described and analyzed the disease burden by age, gender, and year, and created bar and trend charts. We conducted a comparative analysis of the dietary factors influencing these three chronic diseases by generating heatmaps. The joinpoint model was used to analyze the time trends of these three diseases from 1990 to 2021.

**Results:**

From 1990 to 2021, the burden of neoplasms and cardiovascular diseases in China and Japan declined to varying degrees, while the burden of diabetes continued to increase. The main dietary risk factor for neoplasms is a high red meat diet, while for cardiovascular diseases, a high-sodium diet, especially in China. In addition, high meat consumption appears to serve as a protective factor for both Chinese and Japanese populations. Regarding dietary risk factors for diabetes, China is associated with a high red meat diet, whereas Japan is characterized by a high-processed meat diet.

**Conclusion:**

By comparing the burden of chronic diseases related to dietary factors in China and Japan, this study proposes strategies for national healthy diets, such as reducing sodium, processed meat, and red meat intake and increasing whole grains, vegetables, and fruit intake. In addition, attention should be given to the dietary status of the elderly, along with targeted health education initiatives.

## 1 Introduction

Non-communicable diseases (NCDs) are the leading global causes of mortality, accounting for 74% of all deaths (1). The World Health Organization (WHO) classifies NCDs into four categories: cardiovascular diseases (CVDs), neoplasms (cancers), chronic respiratory diseases, and diabetes (2). According to a systematic analysis of the Global Burden of Disease (GBD) Study 2019–2021, NCDs contribute to 1.73 billion disability-adjusted life years (DALYs), becoming the most significant and serious health problem for adults worldwide (3). Moreover, the prevalence of NCDs is likely to increase over time because of population aging, urbanization, and lifestyle changes (4). Relevant data showed that, in 2021, non-communicable diseases were the leading cause of death and disability in the Western Pacific Region. All four non-communicable diseases claimed 12 million lives in the region (5).

Several risk factors are associated with non-communicable diseases; however, modifiable risk factors cover a significant portion. Studies have shown that poor diet is a significant risk factor for non-communicable diseases and impacts the disease burden of NCDs (6). A meta-analysis on the association between a plant-based diet pattern and the risk of type 2 diabetes, cardiovascular disease, cancer, and premature death indicated that maintaining a healthy plant-based dietary pattern is beneficial in reducing the risk of major chronic diseases (7). Another meta-analysis highlighted a consistent and significant positive relationship between ultra-processed food (UPF) intake and the risk of various cancers, including colorectal, breast, and pancreatic cancer (8). An umbrella review of meta-analyses on the association between dietary patterns and the risk of chronic diseases concluded that a healthy dietary pattern may be associated with a lower risk of developing T2D and CVD mortality (9).

China and Japan are two countries in the Western Pacific Region with serious aging populations and an increased burden of chronic diseases. NCDs are major contributors to the health burden, inequalities in health outcomes, and economic burden in China (10). Studies have shown that the burden of NCDs in China is gradually increasing, with mortality rates increasing from 80.0% in 2002 to 88.5% in 2018 (11). A recent study in Japan reported that more than 90% of adults over the age of 75 succumb to a chronic disease, with 80% of individuals experiencing multiple chronic conditions (12). Furthermore, studies revealed that chronic diseases are the leading cause of death in Japan (13, 14). Although China and Japan share similar ethnic and cultural backgrounds, there are distinct differences in population size, composition, and levels of socioeconomic development (15). From 1990 to 2021, China experienced rapid economic growth and significant changes in the lifestyle habits of its population. In contrast, Japan experienced a period of relative social stability during this period. In addition, due to differences in geographical environments, the dietary habits of the two countries also exhibit significant distinctions. In traditional Chinese cuisine, rice and noodles play a dominant role, with a preference for strong flavors, as well as spicy and oily foods being common. On the other hand, the Japanese prefer seafood, vegetables, and tofu, particularly light and delicate cooking styles. These differences between the two countries provide a meaningful comparison.

Our research aimed to compare the similarities and differences in the burden of chronic diseases in China, Japan, and the world, using the latest GBD 2021 data on the prevalence, mortality rates, and disability-adjusted life years (DALYs) of chronic diseases with a specific focus on dietary factors. We hope that this study will provide guidance for reducing disease burden in both countries, gain a deeper understanding of the role of diet in the occurrence and development of chronic diseases in China and Japan, and lay the foundation for the formulation of targeted health policies and intervention measures.

## 2 Methods

### 2.1 Study design

This study used a combination of cross-sectional and longitudinal research methods. Data on annual numbers and age-standardized rates (ASRs) of death and DALYs due to NCDs caused by dietary risk in China, Japan, and the Global from 1990 to 2021 were collected from the GBD 2021 data using the GBD Results Tool.^1^ Previous research fully explained the data collection, processing, and modeling method (16). We selected three major chronic diseases (cardiovascular diseases, neoplasms, and diabetes), as chronic respiratory disease is not captured by dietary risk in the GBD database. We considered dietary risk as risk and death and DALYs as measures.

### 2.2 Dietary risk factor definitions

A dietary risk factor is a behavioral factor causally linked to an increased or decreased probability of getting a disease or injury. Increased probability means the risk factor is dangerous. Fifteen dietary risk factors associated with chronic disease were assessed in the GBD database: “Diet high in processed meat (≥0–4 g/day),” “Diet high in red meat (≥18–27 g/day),” “Diet high in sodium (≥1–5 g/day),” “Diet high in sugar-sweetened beverages (≥50 kCal per 226.8 serving),” “Diet high in trans fatty acids (≥0.0–1.0% of total daily energy),” “Diet low in calcium (≤1.00–1.50 g/day),” “Diet low in fiber (≤19–28 g/day),” “Diet low in fruits (≤200–300 g/day),” “Diet low in legumes (≤50–70 g/day),” “Diet low in nuts and seeds (≤16–25 g/day),” “Diet low in omega-6 Polyunsaturated Fatty Acids (≤9–13% of total daily energy),” “Diet low in seafood omega-3 fatty acids (≤200–300 g/day),” “Diet low in milk (≤350–520 g/day),” “Diet low in vegetables (≤290–430 g/day),” and “Diet low in whole grains (≤100–150 g/day).” The exposure definition and optimal level (or range) of intake have been reported in detail elsewhere (17).

### 2.3 The burden of disease index

The burden of disease index included the summary exposure value (SEV), death, DALY, age-standardized mortality rate (ASMR), age-standardized DALYs rate (ASDR), and age-standardized population attributable fraction (ASPAF). In addition, attributable deaths and DALYs were estimated by multiplying the overall death rate or DALYs by the population attributable fraction (PAF) for each risk–outcome pair, considering factors such as age, sex, cause, and location. The SEV, number, rate, and PAF of diet-related chronic diseases were also directly obtained from the Global Health Data Exchange website.

### 2.4 Statistical analyses

The burden of chronic diseases due to dietary risk factors was analyzed based on age, sex, year, and location. All measures were reported as numbers and ASRs. An estimate of DALYs is computed by summarizing the number of healthy years lost to disease, weighting for severity by disability weights (YLDs), and dividing the number of healthy years lost to disease. Summary exposure value (SEV) measures the risk factor exposure of a population. SEV = 0 signifies no excess risk for the population, and SEV = 1 signifies that the population is at the highest level of risk. According to the GBD 2021 data, the SEV is reported as a risk-weighted prevalence ranging from 0 to 100%. Data were presented as values with a 95% uncertainty interval (UI). We calculated the relative change from 1990 to 2021 using the formula: percentage change (%) = (value in 2021 - value in 1990)/value in 1990 × 100%. The joinpoint regression model assesses time trends for the age-standardized SEV, ASMR, ASDR, and ASPAF. This model divides a straight regression line into several statistically significant trend segments, each described by a linear model. This method aims to identify the optimal number of breakout points to assess significant changes in trends over time (18). Annual percent change (APC), average percent change (AAPC), and 95% confidence intervals (CIs) were calculated using this model. The joinpoint regression analysis was performed using Joinpoint software (version 4.7) from the National Cancer Institute Surveillance Research Program. All statistical analyses were performed using R (version 4.3). GraphPad Prism (version 9.0) was used for plotting. A *p*-value of <0.05 was considered statistically significant.

## 3 Results

### 3.1 Burden of chronic diseases attributable to diet

The age-standardized incidence rate of neoplasms in China in 2021 was 790.17 per 100,000 people, compared to the global incidence rate of 790.33 per 100,000 people. In Japan, the age-standardized incidence rate was 1292.86 per 100,000 people (95% UI: 1125.40, 1504.75). The incidence rate of neoplasms in China was 1814.49 per 100,000 people, while in Japan it was 3303.66 per 100,000 people. The incidence and prevalence rates of cardiovascular disease in China were 811.81 and 6603.72 per 100,000 people, respectively. The incidence and prevalence rates in Japan were 424.23 per 100,000 people and 5040.38 per 100,000 people, respectively. The incidence of diabetes was 244.57 per 100,000 people in China and 284.82 per 100,000 people in Japan. The prevalence rate in China is 5870.91 per 100,000 people, while in Japan it is 6142.29 per 100,000 people (Supplementary Table S1).

From 1990 to 2021, the China’s SEV value decreased by 5.41%, with a percentage change of −13.02%; the age-standardized SEV value decreased by 8.24%, with a percentage change of −19.10%. The Japan’s SEV value decreased by 1.24%, with a percentage change of −2.84%; the age-standardized SEV value decreased by 4.07%, with a percentage change of −9.49% (Table 1). The burden of chronic disease index included population estimated, the PAF, age-standardized PAF, rate and age-standardized mortality rates, and DALYs. For neoplasms, although the estimated number of people in both countries has been increasing, the PAF, mortality rate, and DALYs rate have decreased, and the decrease in China is greater compared to that in Japan. In China, the ASMR decreased by 9.78 per 100,000 people, while in Japan, the ASMR only decreased by 3.29 per 100,000 people. The China’s ASDR decreased by 259.00 per 100,000 people, whereas the Japan’s ASDR decreased by only 85.23 per 100,000 people (ASMR percentage change: China: −53.03% and Japan: -26.52%; ASDR percentage change: China: −55.17% and Japan: −28.64%). For cardiovascular disease, China’s ASMR decreased by 50.86 per 100,000 people, while Japan’s ASMR decreased by 31.38 per 100,000 people. The China’s ASDR decreased by 1131.26 per 100,000 people, whereas the Japan’s ASDR decreased by 547.79 per 100,000 people. While China experienced a greater absolute decrease, Japan exhibited a larger relative decrease in percentage change (ASMR percentage change: China: −39.54% and Japan: -66.91%; ASDR percentage change: China: −43.00% and Japan: −60.29%). On the burden of diabetes, the two countries presented different trends. The ASMR and ASDR in China increased in varying degrees from 1990 to 2021; the ASMR increased by 0.1 per 100,000 people and ASDR by 48.08 per 100,000 people (percentage change: ASMR: 5.83% and ASDR: 56.49%); However, the ASMR in Japan decreased by 1.28 per 100,000 people, while the ASDR increased by 45.71 per 100,000 people (percentage change: ASMR: −67.2% and ASDR: 38.90%) (Table 1).

**Table 1:** The summary exposure values and burden of Neoplasms, Cardiovascular diseases and Diabetes mellitus attributable dietary risks among China, Japan and Global.

**Metric/measure**	**1990 values (95% UI)**			**2021 values (95% UI)**			**Percentage change (%)**		
	**China**	**Japan**	**Global**	**China**	**Japan**	**Global**	**China**	**Japan**	**Global**
**Summary exposure values**									
All ages, %	41.76 (31.3,52.21)	44.00 (32.23,54.71)	39.66 (30.29, 49.33)	36.32 (23.87,48.26)	42.76 (30.49,54.52)	37.73 (28.12, 47.95)	-13.02 (-26.46,0.17)	-2.84 (-13.00,5.31)	-4.86 (-9.29, -0.44)
Age-standardized, %	43.13 (32.56,53.39)	42.85 (31.51,53.54)	40.31 (30.73, 50.09)	34.89 (23.06,47.17)	38.78 (28.36,49.85)	37.62 (28.05, 47.81)	-19.10 (-31.66, -6.12)	-9.49 (-17.7, -3.37)	-6.69 (-10.19, -2.96)
**Neoplasms**									
Death									
Mortality (all ages), 1/10^5^	12.5 (2.53,26.05)	16.41 (4.51,35.86)	8.70 (2.35, 16.22)	12.41 (4.27,25.83)	29.42 (9.13,53.77)	8.49 (2.62, 14.91)	-0.71 (-29.68,46.44)	79.32 (39.22,118.99)	-2.45 (-14.26, 13.68)
ASMR, 1/10^5^	18.44 (3.89,38.01)	12.41 (3.44,27.11)	12.24 (3.32, 22.78)	8.66 (2.98,17.89)	9.12 (2.7,16.61)	7.90 (2.45, 13.85)	-53.03 (-66.42, -32.90)	-26.52 (-43.68, -12.47)	-35.45 (-42.94, -25.38)
DALYs									
All-age rate, 1/10^5^	361.15 (73.85,752.05)	400.35 (103.52,868.77)	232.07 (61.64, 436.11)	309.77 (103.96,640.04)	521.98 (154.27,946.26)	207.87 (62.60, 367.93)	-14.23 (-40,23.66)	30.38 (-0.94,58.13)	-10.43 (-22.3, 3.25)
ASDR, 1/10^5^	469.49 (97.07,975.92)	297.55 (77.24,645.66)	302.48 (80.53, 565.63)	210.49 (70.5,431.87)	212.32 (61.1,381.83)	189.62 (57.13, 335.37)	-55.17 (-68.61, -35.86)	-28.64 (-47.16, -14.72)	-37.31 (-45.38, -27.95)
**Cardiovascular diseases**									
Death									
Mortality (all ages), 1/10^5^	70.54 (40.07,97.69)	57.93 (22.69,87.12)	75.53 (20.90, 107.79)	101.91 (41.81,157.73)	57.20 (19.77,91.82)	73.93 (17.20, 109.76)	44.47 (-3,84.77)	-1.27 (-20.84,9.56)	-2.12 (-12.05, 6.26)
ASMR, 1/10^5^	128.62 (69.7,181.22)	46.90 (18.4,71.04)	113.61 (31.19, 164.63)	77.76 (30.45,121.22)	15.52 (5.26,24.36)	69.81 (16.19, 104.09)	-39.54 (-59.31, -24.82)	-66.91 (-73.32, -63.9)	-38.55 (-44.01, -32.97)
DALYs									
All-age rate, 1/10^5^	1788.28 (1007.52,2447.78)	1183.49 (489.19,1729.99)	1843.62 (507.95, 2550.54)	2127.31 (929.78,3178.72)	939.44 (324.98,1480.92)	1700.34 (412.88, 2443.94)	18.96 (-14.67,50.25)	-20.62 (-37.81, -12.71)	-7.77 (-19.3, -0.78)
ASDR, 1/10^5^	2630.84 (1471.93,3614.8)	908.57 (375.37,1329.07)	2487.47 (675.52, 3480.84)	1499.58 (632.89,2247.22)	360.78 (122.31,544.88)	1563.86 (378.95,2246.75)	-43.00 (-59.68, -28.42)	-60.29 (-69.74, -56.69)	-37.13 (-44.85, -32.23)
**Diabetes mellitus**									
Death									
Mortality (all ages), 1/10^6^	1.07 (0.13,1.9)	2.50 (0.58,4.02)	3.08 (0.58, 4.98)	2.63 (0.30,4.75)	2.07 (0.52,3.36)	4.83 (0.94, 7.87)	145.42 (87.66, 214.22)	-17.32 (-28.54, -7.62)	57.13 (44.98, 67.06)
ASMR, 1/10^5^	1.75 (0.21,3.1)	1.90 (0.44,3.04)	4.55 (0.86, 7.35)	1.85 (0.21,3.34)	0.62 (0.15,0.99)	4.52 (0.88, 7.36)	5.83 (-19.33, 33.1)	-67.20 (-69.49, -64.25)	-0.72 (-7.32, 5.46)
DALYs									
All-age rate, 1/10^5^	65.43 (8.43,118.58)	157.87 (40.26,262.66)	120.93 (23.55, 199.00)	189.94 (23.73,346.3)	332.79 (84.91,576.74)	242.63 (52.55, 404.72)	190.32 (146.98, 228.34)	110.80 (86.77, 135.4)	0.04 (-2.34, 6.70)
ASDR, 1/10^5^	85.11 (10.99,153.97)	117.50 (30.01,195.74)	159.96 (31.17, 262.82)	133.19 (16.98,244.54)	163.21 (0,277.7)	221.34 (47.97, 368.92)	56.49 (33.04, 78.9)	38.90 (23.14, 55.04)	38.38 (28.07, 49.41)

Note: PAF: population-attributable fraction; ASPAF: age-standardized population-attributable fraction; ASMR: age-standardized mortality rate; DALY: disability-adjusted life year; ASDR: age-standardized disability-adjusted life year rate.

### 3.2 A comparison of the age distribution of chronic disease burden attributable to dietary factors in China, Japan, and global

**Figure 1:**
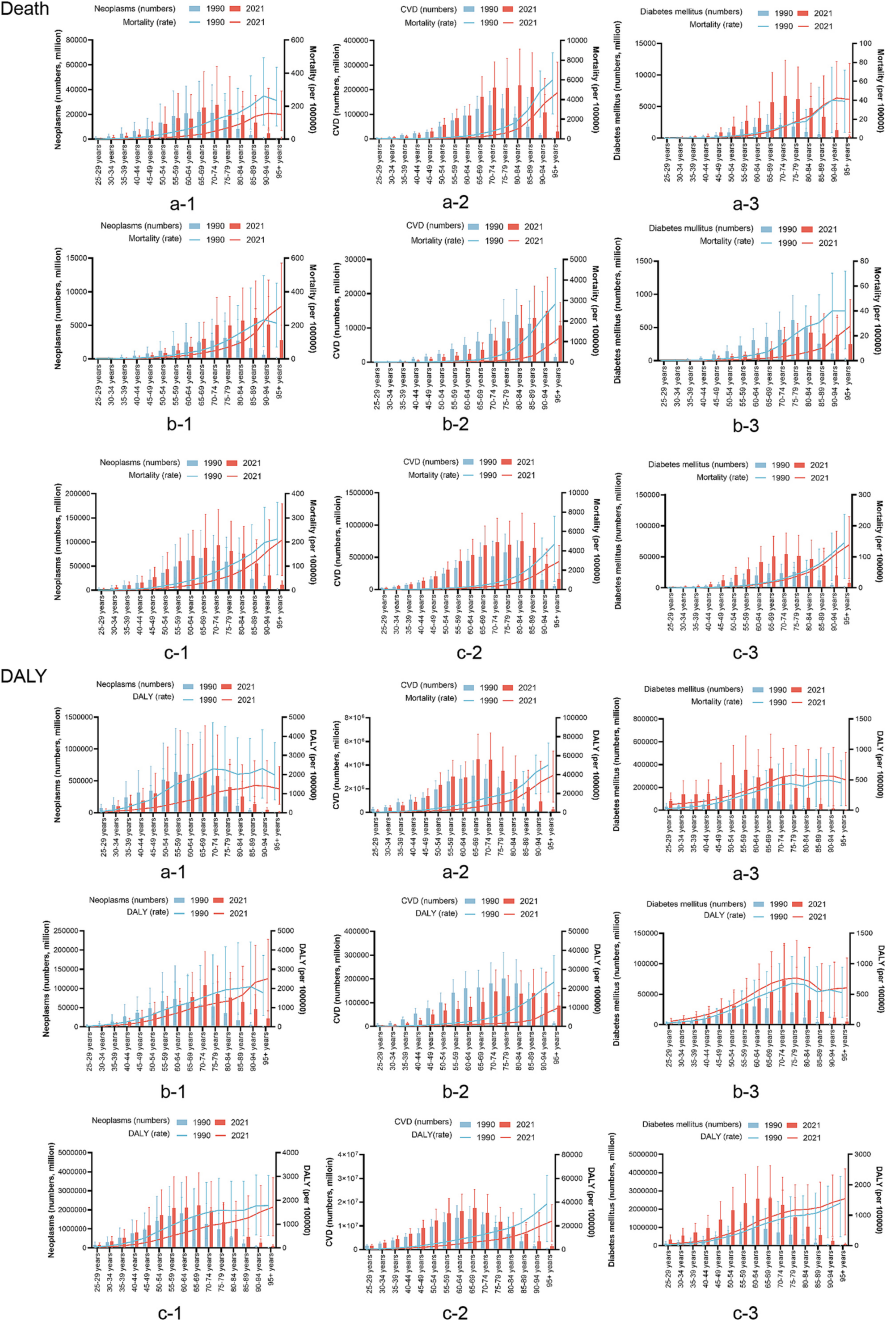
Based on the gender group of a comparison of the age distribution of chronic disease burden attributable to dietary factors in China, Japan, and Global. a: China; b: Japan; c: the global; CVD, cardiovascular disease; DALY, disability-adjusted life years.

Neoplasms, cardiovascular diseases, and diabetes account for the majority of the burden of chronic diseases among the elderly, but there are specific differences in age distribution between China, Japan, and the global. The age distribution of neoplasm-related deaths due to dietary factors and DALYs was similar between China and the global; however, there were differences in distribution between men and women. For cardiovascular disease, the age distribution in China was similar to that of the global, with men exhibiting a younger trend than women, while Japan showed a lower overall age distribution. The age distribution of DALYs attributed to dietary factors for men was similar across China and the global, whereas women were primarily concentrated in the 80–95+ age range. For diabetes, the age distribution of mortality was similar to that of the first two diseases. The age distribution of DALYs for diabetes was similar between China and the world, indicating a younger demographic affected by diabetes-related disabilities; however, DALYs among Japanese men were higher than those among women across all age groups (Figure 1).

**Figure 2:**
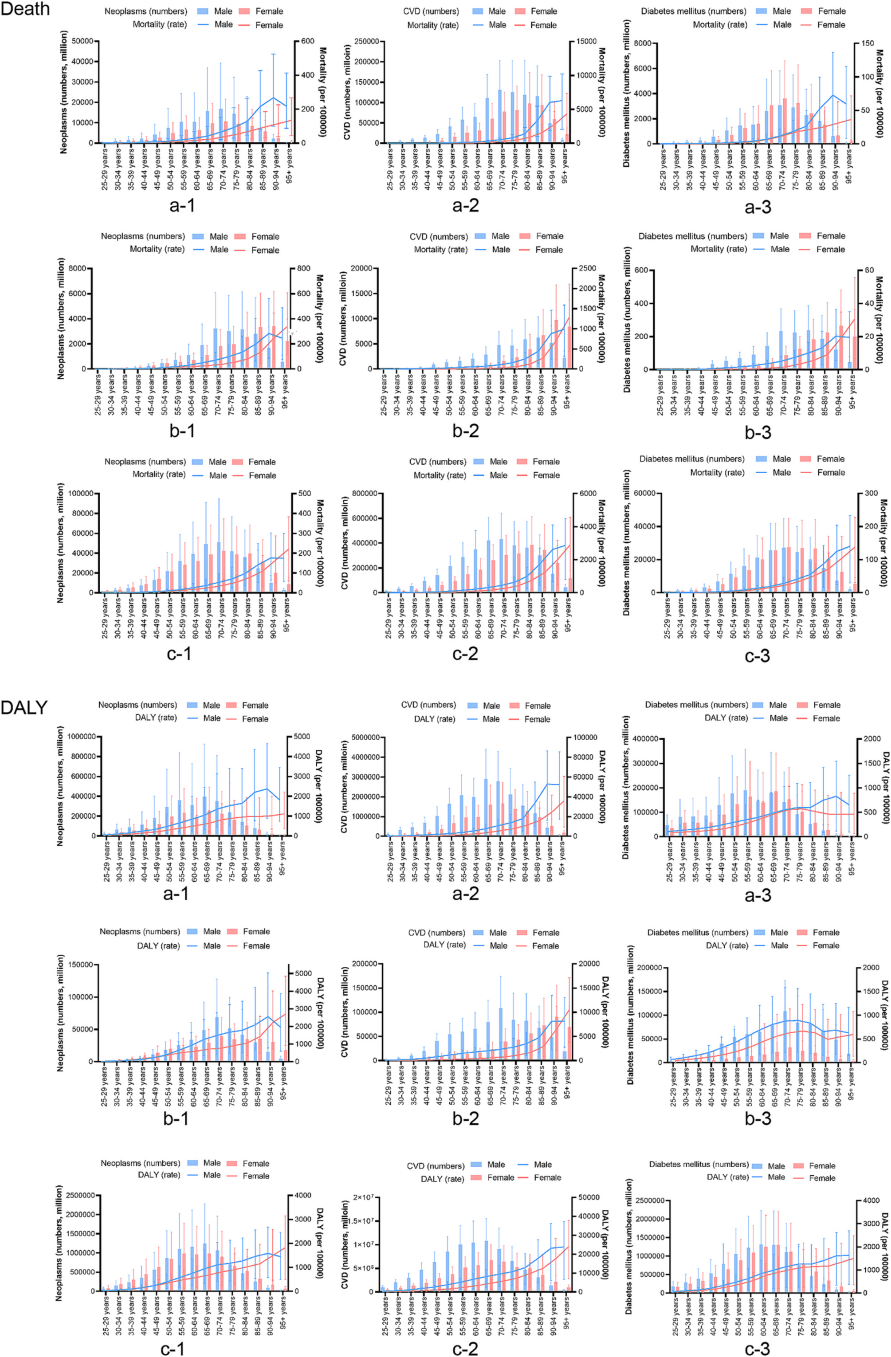
Based on the time group of a comparison of the age distribution of chronic disease burden attributable to dietary factors in China, Japan, and Global. a: China, b: Japan, c: the global, CVD, cardiovascular disease; DALY, disability-adjusted life years.

Compared to 1990, the 2021 disease burden rate of neoplasms in China and the world decreased; however, in Japan, the disease burden increased in the population aged 90 years and over. The burden of CVDs in China, Japan, and the world at all ages has varying degrees of reduction. For diabetes, the mortality rate in Japan decreased significantly in 2021, while there was little change in China and the world. The DALYs rate increased slightly in China, Japan, and the global (Figure 2).

### 3.3 Analysis of dietary factors in the burdens of chronic diseases in China, Japan, and the global

**Figure 3:**
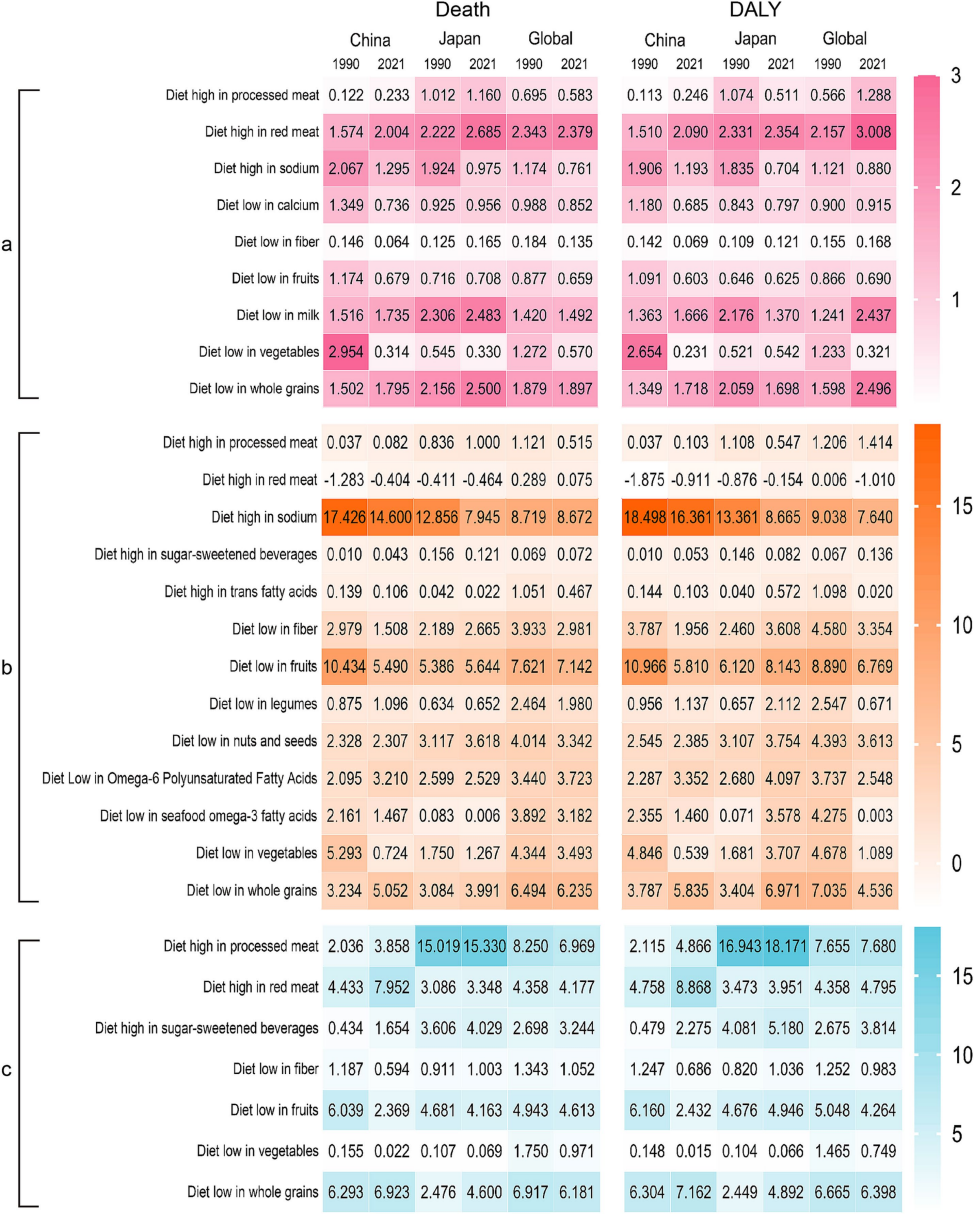
Heatmap of dietary factors in the burdens of chronic diseases in China, Japan, and Global. **(A)** Neoplasms, **(B)** cardiovascular disease, **(C)** diabetes mellitus. DALY, disability-adjusted life years.

There were nine dietary factors associated with neoplasms; however, a “diet high in red meat” has the highest population attributable risk, and the risk increased slightly from 1990 to 2021 in China and Japan. In 1990, “a Diet low in vegetables” was the main dietary risk factor in China, and after 30 years of development, neoplasm deaths attributed to a “Diet low in vegetables” reduced from 2.954 to 0.314%. The ASPAF of DALYs reduced from 2.654 to 0.231% (Figure 3A).

There were 13 dietary factors related to cardiovascular disease, and a “Diet high in sodium” had the highest population attributable risk, especially in China. Although the ASPAF in 2021 has decreased compared with 1990, it is still higher than in Japan and the world. A “Diet low in fruits” is the second largest dietary factor; China’s death attribution percentage decreased from 10.434 to 5.490%, lower than the Japan and world standards. Furthermore, the ASPAF of DALYs in China decreased from 10.966 to 5.816%, while in Japan, it increased from 6.120 to 8.143%. In addition, we also found that a diet high in red meat is a protective factor for Chinese and Japanese populations (Figure 3B).

There are seven dietary factors related to diabetes. China and Japan exhibit varying dietary influences. In China, the dietary factor changed from a “Diet low in whole grains” in 1990 to a “Diet low in red meat” in 2021. On the other hand, Japan has consistently shown a “Diet high in processed meat” and the PAF has slightly increased (Figure 3C).

### 3.4 Temporal trends in chronic diseases attributable to dietary factors in China and Japan from 1990 to 2021

#### 3.4.1 Temporal trends in age-standardized mortality and DALYs for chronic diseases

**Figure 4:**
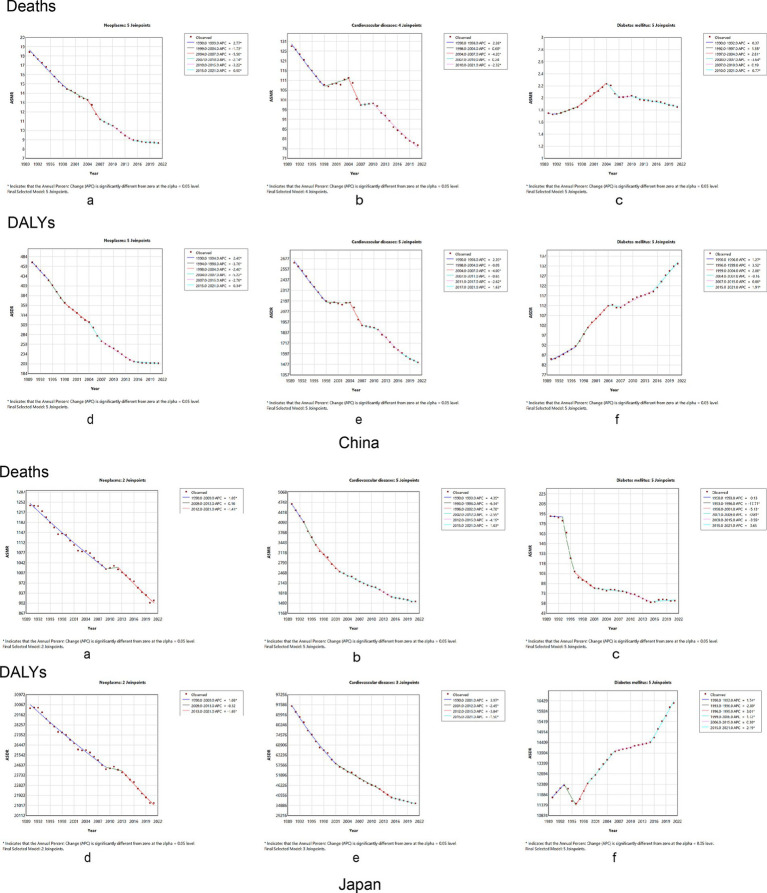
Temporal trends in the burden of chronic diseases attributable to dietary factors from 1990 to 2021 between China and Japan. Neoplasms: **(A)** age-standardized mortality rates, **(B)** age-standardized DALY rates. Cardiovascular disease: **(C)** age-standardized mortality rates, **(D)** age-standardized DALY rates. Diabetes mellitus: **(E)** age-standardized mortality rates, **(F)** age-standardized DALY rates. DALY, disability-adjusted life year.

Figure 4 shows age-standardized mortality and DALYs for chronic diseases in China and Japan, from 1990 to 2021. The AAPC for neoplasms’ ASMR was −2.45 (95% CI: −2.73, −2.17), and the AAPC for the ASDR was −2.57 (95% CI: −2.70, −2.43) in China (Supplementary Table S2). Regression analysis of junction points showed that APC values at each junction point of the neoplasms’ ASMR and ASDR were negative (*p* < 0.05) (Figures 4A,B, China), reflecting the continuous decline in the tumor ASMR and ASDR from 1990 to 2021. The most significant decline occurred from 2004 to 2007. In Japan, the AAPCs of the ASMR and ASDR were −1.04 (95% CI: −1.24, −0.83) and −1.14 (95% CI: −1.27, −1.01), respectively (Supplementary Table S3). In China, the AAPCs of the ASMR and ASDR for cardiovascular diseases were −1.68 (95%CI: −1.98, −1.38) and −1.83 (95% CI: −2.01, −1.65), respectively. In contrast, the AAPCs of the ASMR and ASDR in Japan were −3.53 (95% CI: −3.94, −1.12) and −2.96 (95% CI: −3.22, −2.71), respectively (Supplementary Table S3). According to the time curve of China, the ASMR of cardiovascular diseases marginally decreased at first. The two periods, 1998–2004 and 2007–2010, rose slightly, while the rest of the period was a downward trend; the ASDR of cardiovascular diseases also showed a downward trend (Figures 4C,D, China). Japan’s APC values at each junction point of the ASMR and ASDR were negative (*p* < 0.05) (Figures 4C,D, Japan). The AAPC for the ASMR in diabetes was 0.21 (95% CI: −0.03, 0.44), compared to 1.50 (95% CI: 1.3, 1.69) for the ASDR in China. From 1990 to 2021, the ASMR in diabetes experienced several varying stages of change; however, in general, none were statistically significant (Figure 4E). However, in contrast to the ASMR, the APC in the ASDR in diabetes was positive (*p* < 0.05) (Figure 4F). The ASDR for diabetes has increased annually over the past 30 years. Moreover, the AAPC for the ASMR with diabetes in Japan was negative [−3.47 (95% CI: −4.07, −2.87)], while the AAPC for the ASDR was positive [0.42 (95% CI: 0.40, 0.44)]. Thus, the time-trend model of the ASMR junction point in Japan generally shows a downward trend, while the ASDR model shows an upward trend (Supplementary Table S3).

#### 3.4.2 Temporal trends in the proportion of dietary attributable deaths and DALYs in the age-standardized population for chronic diseases

**Figure 5:**
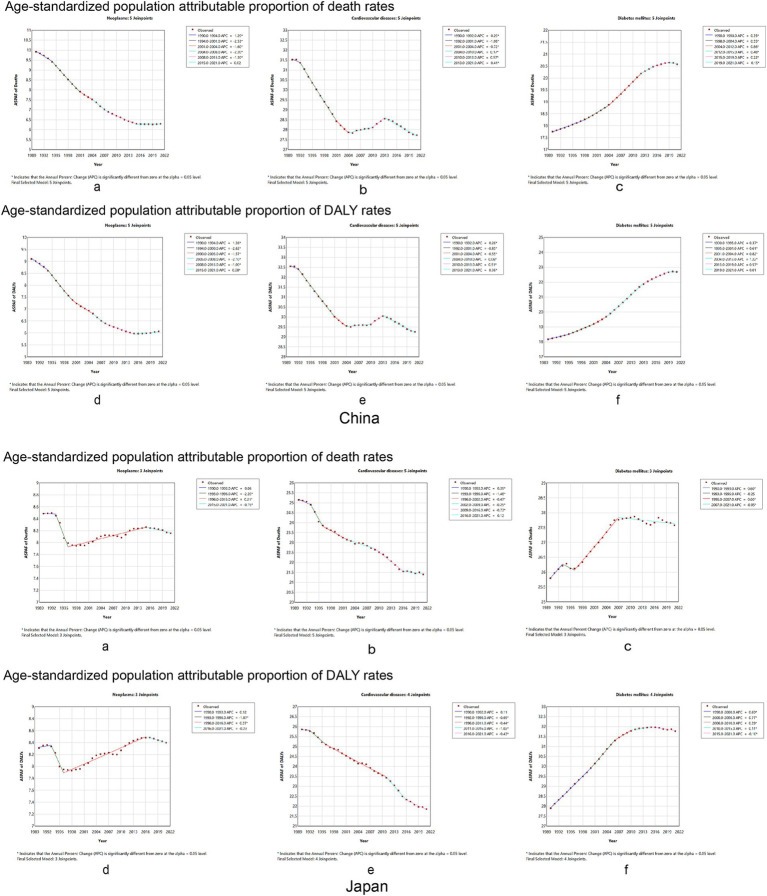
Temporal trends in the global burden of chronic diseases age-standard population attributable proportion rate to dietary factors from 1990 to 2021 between China and Japan. Neoplasms: **(A)** age-standardized population attributable proportion of mortality rates, **(B)** age-standardized population attributable proportion of DALY rates. Cardiovascular disease: **(C)** age-standardized population attributable proportion of mortality rates, **(D)** age-standardized population attributable proportion of DALY rates. Diabetes mellitus: **(E)** age-standardized population attributable proportion of mortality rates, **(F)** age-standardized population attributable proportion of DALY rates. DALY, disability-adjusted life year.

The AAPC and 95% CI of the ASPAF for neoplasm-related death and DALYs rates were −1.47 (95% CI: −1.53, −1.41) and −1.32 (95% CI: −1.37, −1.26) for China (Supplementary Table S2), respectively. Figures 5A,B of China show that the APC of the ASPAF for neoplasm-related death and DALYs rates was negative, similar to mortality and DALYs rates. For Japan, the AAPCs of the ASPAF for death and DALYs rates were −0.13 (95% CI: −0.25, −0.01) and 0.03 (95% CI: −0.12, 0.18) (Supplementary Table S3), respectively. The joinpoint model presented the first decline in the trend of fluctuating results (Figures 5A,B, Japan). The AAPC and 95%CI of the ASPAF for cardiovascular disease and DALYs rates were −0.42 (95% CI: −0.47, −0.37) and −0.35 (95% CI: −0.39, −0.31) for China (Supplementary Table S2), respectively. Figures 5C,D show that the APC with statistically significant ASPAF for both cardiovascular disease death and DALYs was negative. Although there was an upward trend from 2004 to 2013, the overall trend was still declining. The AAPCs of the ASPAF for death and DALYs rates in Japan were −0.52 (95% CI: −0.58, −0.45) and −0.54 (95% CI: −0.60, −0.49), respectively (Supplementary Table S3). The trends were similar to mortality and DALY rates (Figures 5C,D, Japan). The AAPC and 95% CI of the ASPAF for diabetes and DALYs were 0.48 (95% CI: 0.46, 0.50) and 0.72 (95% CI: 0.70, 0.74), respectively, in China (Supplementary Table S2). The ASPAF of death and DALYs trends were increasing (Figures 5E,F, China). In addition, in Japan, the AAPC and 95% CI for the ASPAF and DALYs for diabetes were 0.22 (95% CI: 0.15, 0.29) and 0.42 (95% CI: 0.40, 0.44), respectively (Supplementary Table S3). The joinpoint model shows that the ASPAF of the death time trend rises by volatility, while the ASPAF of DALYs first leveled off after the rising trend (Figures 5E,F, Japan).

## 4 Discussion

The analysis of data from 1990 to 2021 revealed that dietary habits in China and Japan contribute significantly to the burden of chronic diseases. According to the 2021 GBD data, chronic diseases remain a leading health concern in both countries, with neoplasms and diabetes incidence gradually rising. In contrast, CVD trends differ: while the incidence in China is slowly increasing, CVD cases have decreased in Japan. Research has shown that measures to control cardiovascular diseases in Japan began in the 1990s, yielding positive outcomes (19). Japan implemented a universal health program that mandates annual hypertension screenings for individuals over 30. Those identified with severe hypertension and other high-risk conditions are referred to local physicians. In addition, counseling on lifestyle modification is offered by physicians, public health nurses, and dietitians (20).

The study found that age-standardized mortality rates and DALYs due to dietary factors for neoplasms and cardiovascular disease have declined to varying degrees in China and Japan from 1990 to 2021. The age-standardized mortality rates and DALYs for diabetes attributed to dietary factors showed different trends in the two countries. In China, the mortality rate and DALYs for diabetes increased to varying degrees, while the mortality rate in Japan decreased, although the DALYs rate increased. In the Western Pacific Region, the macroeconomic determinants of non-communicable diseases are influenced by unique social, economic, and environmental factors, with rapid aging being one of the most prominent challenges. Developed countries in the West took more than 60 years to transition from aging to an aged society, while China and Japan completed the transition in 23 and 24 years, respectively (21). China has the largest population of elderly individuals aged 65 and above in the world, with a total of 200.6 million people in 2021, accounting for 14.2% of the total population (22). Japan has the highest proportion of elderly individuals, making up 29.3% of its population, a figure that is expected to continue increasing (23). This may partly explain why the diabetes death rate in Japan is declining while the DALYs continue to rise.

Although the main dietary factors contributing to the burden of NCDs differ between the two countries, they can be summarized as a high-sodium diet, high meat consumption, a high-processed meat diet, and a low-grain diet. While the primary dietary factors for neoplasms are similar in China and Japan, their impact is greater in the Japanese population. For cardiovascular diseases, a high-sodium diet is the primary dietary factor among Chinese, whereas, among Japanese, both a high-sodium diet and low fruit intake play a crucial role. Regarding diabetes, China’s primary dietary risk factor is a high-processed meat diet and low whole grain intake, while in Japan, it is a high-processed meat diet. These differences are related to the dietary patterns of each country, which are influenced by factors such as economic development, geographical environment, and cultural practices.

Cohort studies have shown that dietary patterns correlate with NCDs in the Western Pacific Region (24, 25). Research has shown that over the past 30 years, China and Japan have increased consumption of processed and red meat, aligning with global trends (26, 27). Moreover, we found that identical dietary factors have different effects on various diseases. For instance, a high red meat diet is a protective factor against cardiovascular disease in Chinese and Japanese people. In contrast, in Western societies, red meat consumption has been linked to an increased risk of cardiovascular disease; however, moderate consumption of red meat has been negatively correlated with cardiovascular disease risk in the Korean population (28). Similar results have been reported in other population-based studies from Asian countries (29, 30). This phenomenon may arise from differences in dietary patterns between East Asian and Western countries.

Indeed, there are distinct differences in meat consumption among populations in East Asia, Europe, America, and the Near East/North Africa (31). This study defined a high red meat diet as consuming more than 18–27 g per day; however, a meta-analysis found that each additional 100 g of red meat consumed per day is positively associated with an increased risk of all-cause mortality (32). Therefore, whether a red meat diet serves as a protective factor or a risk factor for cardiovascular disease depends on the quantity consumed. For neoplasms and diabetes, a diet high in red meat is considered a major risk factor (33).

In addition, salt intake in China and Japan has declined slightly but remains at approximately 10.0 g/day, which is still the highest in the Western Pacific Region and twice the WHO-recommended limit of less than 5 g/day (33). In addition, vegetable consumption has declined in China and Japan (27, 34). Therefore, specific measures are needed to promote the consumption of minimally processed foods in China and Japan and to develop dietary guidelines for NCD prevention based on geography and local culture. In terms of sodium intake, China’s guideline recommends less than 2000 mg/day (35), which is the same as the WHO/FAO’s recommendation (36). Japan’s dietary guidelines recommend less than 3,000 mg/day for men and less than 2,600 mg/day for women (37). A possible reason for these higher limits may be attributed to the prevalent use of soy sauce, miso, and dried fish products in Japanese cuisine (38).

Despite the existence of various dietary guidelines, many have not effectively adjusted their eating habits, particularly high sodium intake, which continues to pose a significant health risk. There is still considerable progress needed in diet control. To address these challenges, the Chinese government has implemented several national initiatives over the past years, such as the Healthy China 2030 Blueprint, the Healthy China Initiative (2019–2030), and the National Nutrition Plan (2017–2030) (38–40).

Research has shown that increased intake of whole grains is associated with a decrease in NCDs (41). However, China and Japan are experiencing a decline in whole grain intake; therefore, national policies should target promoting the consumption of whole grains. In addition, reducing salt intake is one of the most cost-effective strategies for preventing and managing chronic diseases. Both countries should encourage the reformulation of food products to lower their salt content and promote positive labeling practices (42). To promote healthier eating habits, governments should implement national policies and strategies that enhance public education and awareness while strengthening regulatory oversight of the food industry.

The research used the latest version of the GBD data for analysis and included longitudinal comparisons over time. Based on the results related to chronic diseases and dietary factors in China and Japan, we gained insights into the current disease burden status and trends in both countries. We identified the key dietary factors, which provide crucial guidance for public health policies and interventions, highlighting priority intervention areas and guiding the proper allocation of resources.

This study has some limitations. First, chronic respiratory diseases have been gradually confirmed to be related to dietary factors (43), but the data on dietary risk factors for chronic respiratory diseases have not been independently obtained from the GBD database. Second, the study only explored the relationship between dietary factors included in the GBD database and three chronic diseases. There could be other dietary factors that can impact the occurrence and development of diseases that were not captured in the GBD database. Third, chronic diseases are influenced by multiple factors, yet this study only addressed dietary factors without considering other influencing factors. Finally, the study omitted the analysis of the combined effects of dietary factors on other factors, such as alcohol consumption and smoking.

## 5 Conclusion

The current status and trends in diet-related chronic disease burdens in China and Japan highlight the critical need for the development of targeted policies and strategies. By evaluating the impact of dietary factors on chronic diseases in both countries, we can provide guidance for improving dietary habits, such as reducing sodium intake and increasing the consumption of whole grains. As the population ages, it is important to maintain a balanced diet. Strengthening health education and improving the management of food supply chains are key steps in alleviating the burden of chronic diseases. Although the approaches to the prevention and control of chronic diseases differ between China and Japan, there are valuable lessons each country can learn from the other.

## Data Availability

The datasets presented in this study can be found in online repositories. The names of the repository/repositories and accession number(s) can be found in the article/Supplementary material.

## Footnotes

^1^ http://www.healthdata.org/gbd/
