# Supplementary material for: Burden of diet-related chronic diseases in Chinese and Japanese adults attributable to dietary risk factors from 1990 to 2021: a systematic analysis of the Global Burden of Disease Study 2021
Source: Front Nutr. 2025 Jan 24;11:1472451. doi: 10.3389/fnut.2024.1472451 (PMC11802435; doi:10.3389/fnut.2024.1472451)
Supplement: Supplementary file 1 [file Supplementary_file_1.docx]

**Burden of Diet-Related Chronic Diseases in Chinese and Japanese Adults Attributable to Dietary Risk Factors from 1990 to 2021: A Population-Based Study**

**Catalogue**

[Supplementary Table S1 Changes of incidence and prevalence of three non-communicable diseases in China, Japan and Global from 1990 to 2021 2](#_Toc22883)

[Supplementary Table S2 Joinpoint analysis of temporal trends in the global burden of chronic diseases attributable to dietary risks from 1990 to 2021 in China 5](#_Toc31752)

[Supplementary Table S3 Joinpoint analysis of temporal trends in the global burden of chronic diseases attributable to dietary risks from 1990 to 2021 in Japan 7](#_Toc13674)

**Supplementary Table S1 Changes of incidence and prevalence of three non-communicable diseases in China, Japan and Global from 1990 to 2021**

| **Metric/measure** | **1990 (95% UI)** | | | **2021 (95% UI)** | | | **Percentage change** | | |
| --- | --- | --- | --- | --- | --- | --- | --- | --- | --- |
|  | **China** | **Japan** | **Global** | **China** | **Japan** | **Global** | **China** | **Japan** | **Global** |
| **Neoplasms** |  |  |  |  |  |  |  |  |  |
| **Incidence** |  |  |  |  |  |  |  |  |  |
| All ages Number | 7818410.90  (6478326.81, 9545530.11) | 1894464.73  (1628066.02, 2212827.63) | 34774679.41  (30034912.81, 40425534.96) | 13664748.50  (11787026.26, 15848005.53) | 2364351.28  (2119264.58, 2645563.92) | 66479607.27  (58335731.40, 74980442.95) | 74.78  (56.1, 95.65) | 24.80  (15.8, 34.46) | 91.17  (81.45, 100.07) |
| All ages Rate, 1/10^5^ | 664.57  (550.66, 811.38) | 1505.59  (1293.88, 1758.61) | 651.99  (563.12, 757.94) | 960.45  (828.47, 1113.9) | 1851.53  (1659.6, 2071.74) | 842.44  (739.24, 950.16) | 44.52  (29.08, 61.78) | 22.98  (14.11, 32.50) | 29.21  (22.64, 35.22) |
| All ages Percent, % | 0.19  (0.16, 0.24) | 0.34  (0.29, 0.41) | 0.14  (0.12, 0.16) | 0.31  (0.26, 0.37) | 0.43  (0.38, 0.49) | 0.18  (0.16, 0.21) | 61.28  (43.54, 80.32) | 25.88  (16.04, 35.49) | 33.05  (25.64, 39.43) |
| Age-standardized Rate, 1/10^5^ | 718.73  (608.7, 842.6) | 1316.43  (1127.21, 1552.49) | 758.26  (658.68, 869.22) | 790.17  (676.83, 926.32) | 1292.86  (1125.4, 1504.75) | 790.33  (694.43, 893.01) | 9.94  (2.14, 18.73) | -1.79  (-5.62, 2.1) | 4.23  (1.19, 7.36) |
| Age-standardized Percent, % | 0.21  (0.17, 0.24) | 0.28  (0.23, 0.33) | 0.16  (0.14, 0.19) | 0.25  (0.21, 0.29) | 0.27  (0.23, 0.32) | 0.17  (0.15, 0.19) | 19.71  (11.09, 29.52) | -2.67  (-6.8, 1.28) | 4.03  (0.76, 7.35) |
| **Prevalence** |  |  |  |  |  |  |  |  |  |
| All ages Number | 15599093.58  (13276826.11, 18973574.44) | 4449232.53  (4034091.52, 4949646.33) | 76297772.45  (68878587.77, 85857130.92) | 32811096.43  (29082343.45, 36979938.83) | 6986571.89  (6544960.08, 7417506.52) | 155078828.60  (143578953, 167513892) | 110.34  (83.16, 142.51) | 57.03  (45.00, 68.12) | 103.25  (93.33, 112.77) |
| All ages Rate, 1/10^5^ | 1325.94  (1128.54, 1612.77) | 3535.95  (3206.03, 3933.65) | 1430.51  (1291.4, 1609.73) | 2306.18  (2044.1, 2599.19) | 5471.19  (5125.36, 5808.66) | 1965.17  (1819.45, 2122.75) | 73.93  (51.45, 100.53) | 54.73  (42.88, 65.66) | 37.38  (30.67, 43.81) |
| All ages Percent, % | 1.36  (1.16, 1.66) | 3.73  (3.38, 4.16) | 1.47  (1.33, 1.65) | 2.41  (2.14, 2.72) | 5.74  (5.37, 6.10) | 2.03  (1.89, 2.20) | 76.53  (53.65, 103.41) | 53.93  (42.12, 64.87) | 38.50  (31.76, 44.94) |
| Age-standardized Rate, 1/10^5^ | 1415.07  (1225.85, 1667.63) | 2966.1  (2658.19, 3330.71) | 1694.19  (1550.25, 1881.39) | 1814.49  (1609.18, 2036.94) | 3303.66  (3045.84, 3572.79) | 1828.81  (1692.5, 1975.98) | 28.23  (15.72, 42.54) | 11.38  (5.90, 17.12) | 7.95  (4.09, 11.64) |
| Age-standardized Percent, % | 1.45  (1.26, 1.71) | 3.21  (2.88, 3.60) | 1.73  (1.59, 1.93) | 1.94  (1.72, 2.18) | 3.67  (3.38, 3.97) | 1.90  (1.76, 2.05) | 33.51  (20.5, 48.11) | 14.26  (8.64, 20.15) | 9.60  (5.67, 13.35) |
| **Cardiovascular diseases** | |  |  |  |  |  |  |  |  |
| **Incidence** |  |  |  |  |  |  |  |  |  |
| All ages Number | 6248590.13  (5727577.4, 6853739.37) | 896726.60  (834584.07, 962748.75) | 34742351.23  (31845969.43, 37984785.5) | 16039153.32  (14443324.62, 17780114.96) | 1399672.78  (1289571.86, 1512181.05) | 66809836.88  (60907604.14, 73102151.02) | 156.68  (146.97, 166.87) | 56.09  (49.2, 64.33) | 92.30  (90.09, 94.41) |
| All ages Rate, 1/10^5^ | 531.14  (486.85, 582.57) | 712.66  (663.27, 765.13) | 651.38  (597.08, 712.18) | 1127.34  (1015.17, 1249.7) | 1096.09  (1009.86, 1184.19) | 846.62  (771.83, 926.36) | 112.25  (104.22, 120.67) | 53.80  (47.02, 61.92) | 29.97(28.48, 31.40) |
| All ages Percent, % | 0.15  (0.14, 0.17) | 0.16  (0.15, 0.18) | 0.14  (0.12, 0.15) | 0.36  (0.33, 0.41) | 0.26  (0.23, 0.28) | 0.18  (0.16, 0.20) | 136.86  (125.49, 148.18) | 57.43  (49.48, 66.37) | 33.84  (31.19, 36.71) |
| Age-standardized Rate, 1/10^5^ | 783.90  (718.42, 857.63) | 540.06  (505.07, 578.35) | 878.79  (808.17, 959.9) | 811.81  (736.14, 892.01) | 424.23  (395.92, 454.32) | 787.04  (719.74, 859.70) | 3.56  (1.30, 5.85) | -21.45  (-23.6, -19.29) | -10.44  (-11.49, -9.42) |
| Age-standardized Percent, % | 0.22  (0.20, 0.25) | 0.11  (0.10, 0.13) | 0.19  (0.17, 0.21) | 0.25  (0.23, 0.28) | 0.09  (0.08, 0.10) | 0.17  (0.15, 0.19) | 12.76  (9.77, 15.7) | -22.15  (-24.41, -19.77) | -10.61  (-12.01, -9.13) |
| **Prevalence** |  |  |  |  |  |  |  |  |  |
| All ages Number | 52364490.16  (48335271.83, 55935626.22) | 9298353.40  (8764278.68, 9849708.14) | 289550232  (270659430.7, 305371954.4) | 133845554.6  (123702970.8, 144518967.8) | 16236872.70  (15324471.07, 17204882.67) | 612059321.60  (570319527.8, 649806888.8) | 155.60  (145.77, 166.02) | 74.62  (69.75, 80.64) | 111.38  (107.59, 115.51) |
| All ages Rate, 1/10^5^ | 4451.03  (4108.54, 4754.58) | 7389.71  (6965.27, 7827.89) | 5428.77  (5074.59, 5725.41) | 9407.55  (8694.66, 10157.75) | 12715.11  (12000.61, 13473.16) | 7756.08  (7227.14, 8234.42) | 111.36  (103.22, 119.97) | 72.06  (67.26, 78) | 42.87  (40.31, 45.66) |
| All ages Percent, % | 4.58  (4.22, 4.88) | 7.80  (7.33, 8.26) | 5.57  (5.21, 5.89) | 9.82  (9.08, 10.63) | 13.35  (12.6, 14.14) | 8.03  (7.48, 8.53) | 114.51  (106.21, 123.51) | 71.18  (66.18, 76.97) | 44.04  (41.47, 46.95) |
| Age-standardized Rate, 1/10^5^ | 6024.24  (5599.03, 6393.02) | 5568.89  (5253.32, 5881.71) | 7116.05  (6668.14, 7482.02) | 6603.72  (6121.9, 7087.64) | 5040.38  (4708.92, 5378.6) | 7178.73  (6696.15, 7620.67) | 9.62  (7.28, 12.69) | -9.49  (-12.03, -6.58) | 0.88  (-0.64, 2.69) |
| Age-standardized Percent, % | 6.19  (5.76, 6.59) | 6.03  (5.68, 6.36) | 7.28  (6.82, 7.66) | 7.07  (6.55, 7.60) | 5.59  (5.26, 5.96) | 7.46  (6.96, 7.93) | 14.13  (11.48, 17.35) | -7.15  (-9.9, -4.16) | 2.43  (0.88, 4.25) |
| **Diabetes mellitus** |  |  |  |  |  |  |  |  |  |
| **Incidence** |  |  |  |  |  |  |  |  |  |
| All ages Number | 1836952.65  (1648299.74, 2052189.57) | 308478.45  (273254.64, 345115.57) | 7780271.86  (7193590.95, 8397763.49) | 4003543.82  (3603717.14, 4441554.24) | 511919.64  (454851.54, 574251.45) | 24442180.04  (22643512.77, 26301698.29) | 117.94  (103.51, 132.54) | 65.95  (56.48, 76.22) | 214.16  (207.16, 221.45) |
| All ages Rate, 1/10^5^ | 156.14  (140.11, 174.44) | 245.16  (217.16, 274.27) | 145.87  (134.87, 157.45) | 281.40  (253.29, 312.18) | 400.88  (356.19, 449.7) | 309.73  (286.94, 333.3) | 80.22  (68.28, 92.29) | 63.52  (54.19, 73.64) | 112.33  (107.6, 117.27) |
| All ages Percent, % | 0.04  (0.04, 0.05) | 0.06  (0.05, 0.06) | 0.03  (0.03, 0.03) | 0.09  (0.08, 0.10) | 0.09  (0.08, 0.11) | 0.07  (0.06, 0.07) | 101.09  (86.9, 116.06) | 67.38  (57.25, 78.6) | 118.64  (112.9, 125.51) |
| Age-standardized Rate, 1/10^5^ | 163.34  (144.74, 181.98) | 191.06  (170.36, 213.3) | 167.65  (153.97, 181.03) | 244.57  (223.72, 266.48) | 284.82  (255.61, 317.73) | 287.31  (266.94, 308.83) | 49.73  (43.16, 58.82) | 49.07  (43.18, 53.94) | 71.37  (67.82, 74.5) |
| Age-standardized Percent, % | 0.05  (0.04, 0.05) | 0.04  (0.04, 0.05) | 0.04  (0.03, 0.04) | 0.08  (0.07, 0.08) | 0.06  (0.05, 0.07) | 0.06  (0.06, 0.07) | 63.02  (54.94, 73.53) | 47.74  (41.61, 52.81) | 71.05  (67.17, 74.98) |
| **Prevalence** |  |  |  |  |  |  |  |  |  |
| All ages Number | 35352298.81  (31762274.65, 39043128.81) | 6075397.34  (5482714.69, 6753551.08) | 139107876.90  (128055501.20, 150839975.40) | 117288553.90  (107649694.5, 128071007.5) | 14845186.84  (13437498.15, 16453408.79) | 525654113.30  (490915907.3, 565380792.3) | 231.77  (216.77, 251.1) | 144.35  (133.88, 153.5) | 277.88  (269.98, 286.4) |
| All ages Rate, 1/10^5^ | 3004.98  (2699.82, 3318.7) | 4828.32  (4357.3, 5367.27) | 2608.13  (2400.91, 2828.1) | 8243.82  (7566.33, 9001.68) | 11625.28  (10522.92, 12884.68) | 6661.14  (6220.93, 7164.56) | 174.34  (161.94, 190.32) | 140.77  (130.45, 149.79) | 155.40  (150.06, 161.16) |
| All ages Percent, % | 3.09  (2.78, 3.41) | 5.09  (4.59, 5.67) | 2.68  (2.46, 2.90) | 8.60  (7.88, 9.40) | 12.20  (11.04, 13.55) | 6.89  (6.43, 7.41) | 178.44  (166.04, 194.96) | 139.53  (129.23, 148.46) | 157.49  (152, 163.35) |
| Age-standardized Rate, 1/10^5^ | 3581.17  (3197.11, 3968.53) | 3668.79  (3321.93, 4058.17) | 3215.75  (2962.07, 3498.9) | 6142.29  (5601.11, 6704.38) | 5870.91  (5273.67, 6446.93) | 6123.59  (5723.41, 6585.82) | 71.52  (61.76, 83.68) | 60.02  (54.19, 64.78) | 90.43  (85.98, 95.31) |
| Age-standardized Percent, % | 3.68  (3.29, 4.08) | 3.97  (3.59, 4.40) | 3.29  (3.03, 3.58) | 6.57  (6.00, 7.17) | 6.52  (5.86, 7.16) | 6.36  (5.94, 6.84) | 78.58  (68.33, 91.32) | 64.16  (58.28, 69.2) | 93.35  (89.05, 98.22) |

**Supplementary Table S2 Joinpoint analysis of temporal trends in the global burden of chronic diseases attributable to dietary risks from 1990 to 2021 in China**

| **Metric** | **Trend 1** | | **Trend 2** | | **Trend 3** | | **Trend 4** | | **Trend 5** | | **Trend 6** | | **1990-2021** |
| --- | --- | --- | --- | --- | --- | --- | --- | --- | --- | --- | --- | --- | --- |
|  | **Period** | **APC, ^a^% (95% CI)** | **Period** | **APC, % (95% CI)** | **Period** | **APC, %**  **(95% CI)** | **Period** | **APC, % (95% CI)** | **Period** | **APC, % (95% CI)** | **Period** | **APC, % (95% CI)** | **AAPC, ^b^ % (95% CI)** |
| Age-standardized mortality rate | | |  |  |  |  |  |  |  |  |  |  |  |
| Neoplasms | 1990-  1999 | -2.77  (-2.94, -2.60) | 1999-  2004 | -1.73  (-2.34, -1.11) | 2004-  2007 | -5.56  (-7.39, -3.68) | 2007-  2010 | -2.14  (-4.04, -0.21) | 2010-  2015 | -3.22  (-3.81, -2.63) | 2015-  2021 | -0.50  (-0.81, -0.18) | -2.45  (-2.73, -2.17) |
| Cardiovascular diseases | 1990-  1998 | -2.26  (-2.46, -2.06) | 1998-  2004 | 0.60  (-0.14, 1.07) | 2004-  2007 | -4.20  (-6.29, -2.07) | 2007-  2010 | 0.24  (-1.97, 2.5) | 2010-  2021 | -2.32  (-2.47, -2.16) |  |  | -1.68  (-1.98, -1.38) |
| Diabetes mellitus | 1990-  1992 | -0.37  (-1.89, 1.18) | 1992-  1997 | 1.38  (-0.89, 1.86) | 1997-  2004 | 2.81  (-2.54, 3.07) | 2004-  2007 | -3.64  (-5.14, -2.12) | 2007-  2010 | 0.19  (-1.37, 1.78) | 2010-  2021 | -0.77  (-0.88, -0.67) | 0.21  (-0.03, 0.44) |
| Age-standardized population attributable proportion of death rates | | | | | |  |  |  |  |  |  |  |  |
| Neoplasms | 1990-  1994 | -1.29  (-1.44, -1.15) | 1994-  2001 | -2.53  (-2.61, -2.46) | 2001-  2004 | -1.60  (-2.06, -1.15) | 2004-  2008 | -2.20  (-2.42, -1.97) | 2008-  2015 | -1.3  (-1.38, -1.23) | 2015-  2021 | 0.02  (-0.06, 0.09) | -1.47  (-1.53, -1.41) |
| Cardiovascular diseases | 1990-  1992 | -0.29  (-0.54, -0.03) | 1992-  2001 | -1.08  (-1.11, -1.05) | 2001-  2004 | -0.72  (-1.02, -0.42） | 2004-  2010 | 0.17  (0.10,0.24) | 2010-  2013 | 0.57  (0.25, 0.90) | 2013-  2021 | -0.41  (-0.44, -0.37) | -0.42  (-0.47, -0.37) |
| Diabetes mellitus | 1990-  1998 | 0.35  (0.33, 0.37) | 1998-  2004 | 0.55  (0.51, 0.58) | 2004-  2012 | 0.86  (0.84, 0.88) | 2012-  2015 | 0.48  (0.33, 0.63) | 2015-  2019 | 0.22  (0.14, 0.29) | 2019-  2021 | -0.19  (-0.34, -0.04) | 0.48  (0.46, 0.50) |
| Age-standardized DALY ^c^ rate | | |  |  |  |  |  |  |  |  |  |  |  |
| Neoplasms | 1990-  1994 | -2.49  (-2.83, -2.15) | 1994-  1998 | -3.76  (-4.27, -3.25) | 1998-  2004 | -2.40  (-2.64, -2.16) | 2004-  2007 | -5.22  (-6.24, -4.18) | 2007-  2015 | -2.78  (-2.92, -2.64) | 2015-  2021 | -0.34  (-0.52, -0.16) | -2.57  (-2.70, -2.43) |
| Cardiovascular diseases | 1990-  1998 | -2.39  (-2.52, -2.26) | 1998-  2004 | -0.09  (-0.38, 0.21) | 2004-  2007 | -4.00  (-5.32, -2.67) | 2007-  2011 | -0.65  (-1.36, 0.06) | 2011-  2017 | -2.62  (-2.93, -2.3) | 2017-  2021 | -1.63  (-2.10, -1.15) | -1.83  (-2.01, -1.65) |
| Diabetes mellitus | 1990-  1996 | 1.27  (1.05, 1.5) | 1996-  1999 | 3.52  (2.17, 4.88) | 1999-  2004 | 2.08  (1.66, 2.51) | 2004-  2007 | -0.16  (-1.48, 1.18) | 2007-  2015 | 0.88  (0.70,1.06) | 2015-  2021 | 1.91  (1.68, 2.15) | 1.50  (1.31, 1.69) |
| **Age-standardized population attributable proportion of DALY rates** | | | | |  |  |  |  |  |  |  |  |  |
| Neoplasms | 1990-  1994 | -1.36  (-1.5, -1.23) | 1994-  2000 | -2.62  (-2.72, -2.52) | 2000-  2005 | -1.57  (-1.71, -1.43) | 2005-  2008 | -2.10  (-2.53, -1.67) | 2008-  2015 | -1.00  (-1.07, -0.93) | 2015-  2021 | 0.28  (0.21, 0.35) | -1.32  (-1.37, -1.26) |
| Cardiovascular diseases | 1990-  1992 | -0.26  (-0.49, -0.03) | 1992-  2001 | -0.85  (-0.87, -0.82) | 2001-  2004 | -0.55  (-0.82, -0.29) | 2004-  2010 | 0.06  (0.00, 0.13) | 2010-  2013 | 0.51  (0.22, 0.80) | 2013-  2021 | -0.36  (-0.4, -0.33) | -0.35  (-0.39, -0.31) |
| Diabetes mellitus | 1990-  1995 | 0.37  (0.34, 0.40) | 1995-  2001 | 0.61  (0.58, 0.63) | 2001-  2004 | 0.82  (0.70, 0.95) | 2004-  2013 | 1.22  (1.2, 1.23) | 2013-  2019 | 0.57  (0.54, 0.60) | 2019-  2021 | 0.01  (-0.11, 0.14) | 0.72  (0.70, 0.74) |

0.48

**Supplementary Table S3 Joinpoint analysis of temporal trends in the global burden of chronic diseases attributable to dietary risks from 1990 to 2021 in Japan**

| **Metric** | **Trend 1** | | **Trend 2** | | **Trend 3** | | **Trend 4** | | **Trend 5** | | **Trend 6** | | **1990-2021** |
| --- | --- | --- | --- | --- | --- | --- | --- | --- | --- | --- | --- | --- | --- |
|  | **Period** | **APC, ^a^% (95% CI)** | **Period** | **APC, %**  **(95% CI)** | **Period** | **APC, % (95% CI)** | **Period** | **APC, % (95% CI)** | **Period** | **APC, % (95% CI)** | **Period** | **APC, % (95% CI)** | **AAPC, ^b^ % (95% CI)** |
| Age-standardized mortality rate | | |  |  |  |  |  |  |  |  |  |  |  |
| Neoplasms | 1990-  2009 | -1.05  (-1.11, -0.98) | 2009-  2012 | 0.16  (-1.98, 2.34) | 2012-  2021 | -1.41  (-1.60, -1.22) |  |  |  |  |  |  | -1.04  (-1.24, -0.83) |
| Cardiovascular diseases | 1990-  1993 | -4.35  (-5.68, -3.01) | 1993-  1996 | -6.34  (-8.97, -3.64) | 1996-  2002 | -4.78  (-5.40, -4.16) | 2002-  2012 | -2.59  (-2.86, -2.32) | 2012-  2015 | -4.19  (-7.12, -1.17) | 2015-  2021 | -1.63  (-2.14, -1.12) | -3.53  (-3.94, -3.11) |
| Diabetes mellitus | 1990-  1993 | -0.13  (-2.60, 2.40) | 1993-  1996 | -17.71  (-21.75, -13.47) | 1996-  2001 | -5.13  (-6.62, -3.63) | 2001-  2009 | -0.81  (-1.46, -0.16) | 2009-  2015 | -3.55  (-4.60, -2.48) | 2015-  2021 | 0.65  (-0.18, 1.48) | -3.47  (-4.07, -2.87) |
| **Age-standardized population attributable proportion of death rates** | | | | | |  |  |  |  |  |  |  |  |
| Neoplasms | 1990-  1993 | -0.06  (-0.63, 0.51) | 1993-  1996 | -2.20  (-3.28, -1.11) | 1996-  2015 | 0.21  (0.18, 0.25) | 2015-  2021 | -0.19  (-0.35, -0.03) |  |  |  |  | -0.13  (-0.25, -0.01) |
| Cardiovascular diseases | 1990-  1993 | -0.35  (-0.61, -0.09) | 1993-  1996 | -1.48  (-2.00, -0.95) | 1996-  2002 | -0.47  (-0.60, -0.35) | 2002-  2009 | -0.29  (-0.38, -0.19) | 2009-  2016 | -0.73  (-0.82, -0.63) | 2016-  2021 | -0.12  (-0.25, 0.00) | -0.52  (-0.58, -0.45) |
| Diabetes mellitus | 1990-  1993 | 0.60  (0.27, 0.93) | 1993-  1996 | -0.25  (-0.90, 0.41) | 1996-  2007 | 0.60  (0.55, 0.65) | 2007-  2021 | -0.05  (-0.08, -0.02) |  |  |  |  | 0.22  (0.15, 0.29) |
| **Age-standardized DALYc rate** | | |  |  |  |  |  |  |  |  |  |  |  |
| Neoplasms | 1990-  2009 | -1.08  (-1.14, -1.02) | 2009-  2013 | -0.32  (-1.25, 0.62) | 2013-  2021 | -1.69  (-1.89, -1.5) |  |  |  |  |  |  | -1.14  (-1.27, -1.01) |
| Cardiovascular diseases | 1990-  2001 | -3.97  (-4.12, -3.82) | 2001-  2012 | -2.49  (-2.68, -2.3) | 2012-  2015 | -3.84  (-6.30, -1.32) | 2015-  2021 | -1.51  (-1.93, -1.08) |  |  |  |  | -2.96  (-3.22, -2.71) |
| Diabetes mellitus | 1990-  1993 | 1.74  (1.33, 2.15) | 1993-  1996 | -2.80  (-3.59, -2.01) | 1996-  1999 | 3.01  (2.17, 3.86) | 1999-  2006 | 1.72  (1.58, 1.87) | 2006-  2015 | 0.30  (0.22, 0.39) | 2015-  2021 | 2.19  (2.05, 2.33) | 1.08  (0.96, 1.20) |
| **Age-standardized population attributable proportion of DALY rates** | | | | |  |  |  |  |  |  |  |  |  |
| Neoplasms | 1990-1993 | 0.12  (-0.58, 0.83) | 1993-1996 | -1.87  (-3.2, -0.53) | 1996-  2016 | 0.37  (0.33, 0.41) | 2016-  2021 | -0.23  (-0.48, 0.03) |  |  |  |  | 0.03  (-0.12, 0.18) |
| Cardiovascular diseases | 1990-1992 | -0.11  (-0.64, 0.42) | 1992-1996 | -0.69  (-0.95, -0.43) | 1996-  2011 | -0.44  (-0.47, -0.42) | 2011-  2016 | -1.02  (-1.20, -0.83) | 2016-  2021 | -0.43  (-0.56, -0.3) |  |  | -0.54  (-0.60, -0.49) |
| Diabetes mellitus | 1990-2000 | 0.69  (0.68, 0.71) | 2000-2006 | 0.77  (0.73, 0.81) | 2006-  2010 | 0.39  (0.31, 0.47) | 2010-  2015 | 0.11  (0.06, 0.16) | 2015-  2021 | -0.10  (-0.13, -0.08) |  |  | 0.42  (0.40, 0.44) |

Note: a: APC: Annual percentage change, b: AAPC: Average annual percentage change, c: DALY: Disability Adjusted of Life Year.
